# *N*,*N*′-Bis(pyridin-2-yl)benzene-1,4-dicarboxamide

**DOI:** 10.1107/S1600536810051172

**Published:** 2010-12-11

**Authors:** Ta-Pin Tsai, Hui-Lin Hsiao, Jhy-Der Chen

**Affiliations:** a Department of Chemistry, Chung-Yuan Christian University, Chung-Li, Taiwan

## Abstract

Mol­ecules of the title compound, C_18_H_14_N_4_O_2_, are located around an inversion center and connected into chains in the crystal *via* inter­molecular N—H⋯N hydrogen bonds generating an *R*_2_^2^(8) motif.

## Related literature

For *N*,*N′*-bis­(pyridin­yl) derivatives of 1,4-benzene­dicarbox­amide and their metal complexes, see: Tsai *et al.* (2010 ▶).
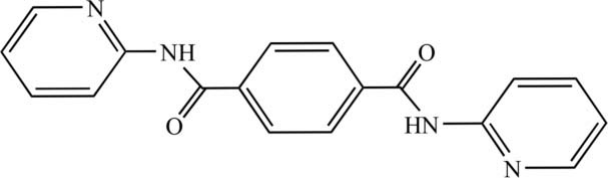


## Experimental

### 

#### Crystal data

C_18_H_14_N_4_O_2_
*M* *_r_* = 318.33
Triclinic, 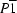

*a* = 5.7895 (4) Å
*b* = 7.8315 (6) Å
*c* = 8.8460 (5) Å
α = 82.906 (6)°
β = 74.083 (5)°
γ = 73.695 (6)°
*V* = 369.72 (4) Å^3^
*Z* = 1
Mo *K*α radiation
μ = 0.10 mm^−1^
*T* = 298 K
0.60 × 0.60 × 0.56 mm

#### Data collection

Siemens P4 diffractometer
Absorption correction: ψ scan (*XSCANS*; Siemens, 1995 ▶) *T*_min_ = 0.831, *T*_max_ = 0.851
1962 measured reflections
1787 independent reflections
1521 reflections with *I* > 2σ(*I*)
*R*_int_ = 0.013
3 standard reflections every 97 reflections  intensity decay: none

#### Refinement

*R*[*F* ^2^ > 2σ(*F* ^2^)] = 0.040
*wR*(*F* ^2^) = 0.112
*S* = 1.06
1787 reflections
110 parameters
H-atom parameters constrained
Δρ_max_ = 0.32 e Å^−3^
Δρ_min_ = −0.19 e Å^−3^

### 

Data collection: *XSCANS* (Siemens, 1995 ▶); cell refinement: *XSCANS*; data reduction: *XSCANS* and *SHELXTL* (Sheldrick, 2008 ▶); program(s) used to solve structure: *SHELXS97* (Sheldrick, 2008 ▶); program(s) used to refine structure: *SHELXL97* (Sheldrick, 2008 ▶); molecular graphics: *SHELXTL*; software used to prepare material for publication: *SHELXTL*.

## Supplementary Material

Crystal structure: contains datablocks I, global. DOI: 10.1107/S1600536810051172/gk2331sup1.cif

Structure factors: contains datablocks I. DOI: 10.1107/S1600536810051172/gk2331Isup2.hkl

Additional supplementary materials:  crystallographic information; 3D view; checkCIF report

## Figures and Tables

**Table 1:** Hydrogen-bond geometry (Å, °)

*D*—H⋯*A*	*D*—H	H⋯*A*	*D*⋯*A*	*D*—H⋯*A*
N1—H1*A*⋯N2^i^	0.86	2.34	3.1679 (15)	163

Symmetry code: (i) 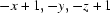.

**Fig. 1.**
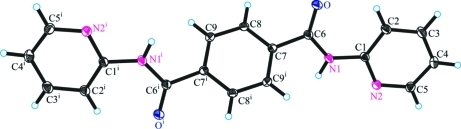
Crystal structure of the title compound with atom labeling and displacement ellipsoids drawn at the 30% probability level. Symmetry code: (i) =-x + 2,-y,-z.

## supplementary crystallographic information

### Comment

Several Cu(II), Cd(II) and Hg(II) complexes containg *N*,*N'*-bis(2/3-aryl)-1,4-benzenedicarboxamide ligands have been reported, which show one-dimensional and two-dimensional structures (Tsai, *et al.*, 2010). Within this project the crystal structure of the title compound was determined.

In its crystal structure intermolecular N—H···N hydrogen bonds are found (Tab. 1) and the molecule is located on a center of inversion (Fig. 1).

### Experimental

The title compound was prepared according to a published procedure (Tsai, *et al.*, 2010). Block crystals suitable for X-ray crystallography were obtained by slow evaporization of the solvent from a solution of the title compound in methanol.

### Refinement

All the hydrogen atoms were placed into idealized positions and refined in the riding atom approximation with *C*—H = 0.93 Å, N—H = 0.86 Å and *U*_iso_(H) = 1.2 *U*_eq_(C, N).

### Crystal data

**Table:**

C_18_H_14_N_4_O_2_	*Z* = 1
*M**_r_* = 318.33	*F*(000) = 166
Triclinic, *P*1	*D*_x_ = 1.430 Mg m^−^^3^
Hall symbol: -P 1	Mo *K*α radiation, λ = 0.71073 Å
*a* = 5.7895 (4) Å	Cell parameters from 50 reflections
*b* = 7.8315 (6) Å	θ = 4.8–15.0°
*c* = 8.8460 (5) Å	µ = 0.10 mm^−^^1^
α = 82.906 (6)°	*T* = 298 K
β = 74.083 (5)°	Block, pale yellow
γ = 73.695 (6)°	0.60 × 0.60 × 0.56 mm
*V* = 369.72 (4) Å^3^	

### Data collection

**Table:**

Bruker P4 diffractometer	1521 reflections with *I* > 2σ(*I*)
Radiation source: fine-focus sealed tube	*R*_int_ = 0.013
graphite	θ_max_ = 28.0°, θ_min_ = 2.4°
ω scans	*h* = 0→7
Absorption correction: ψ scan (*XSCANS*; Siemens, 1995)	*k* = −9→10
*T*_min_ = 0.831, *T*_max_ = 0.851	*l* = −11→11
1962 measured reflections	3 standard reflections every 97 reflections
1787 independent reflections	intensity decay: none

### Refinement

**Table:**

Refinement on *F*^2^	Secondary atom site location: difference Fourier map
Least-squares matrix: full	Hydrogen site location: inferred from neighbouring sites
*R*[*F*^2^ > 2σ(*F*^2^)] = 0.040	H-atom parameters constrained
*wR*(*F*^2^) = 0.112	*w* = 1/[σ^2^(*F*_o_^2^) + (0.0468*P*)^2^ + 0.1049*P*] where *P* = (*F*_o_^2^ + 2*F*_c_^2^)/3
*S* = 1.06	(Δ/σ)_max_ < 0.001
1787 reflections	Δρ_max_ = 0.32 e Å^−^^3^
110 parameters	Δρ_min_ = −0.19 e Å^−^^3^
0 restraints	Extinction correction: *SHELXL97* (Sheldrick, 2008), Fc^*^=kFc[1+0.001xFc^2^λ^3^/sin(2θ)]^-1/4^
Primary atom site location: structure-invariant direct methods	Extinction coefficient: 0.193 (17)

### Special details

**Table:**

Experimental. Refinement of *F*^2^ against ALL reflections. The weighted *R*-factor *wR* and goodness of fit *S* are based on *F*^2^, conventional *R*-factors *R* are based on *F*, with *F* set to zero for negative *F*^2^. The threshold expression of *F*^2^ > σ(*F*^2^) is used only for calculating *R*-factors(gt) *etc*. and is not relevant to the choice of reflections for refinement. *R*-factors based on *F*^2^ are statistically about twice as large as those based on *F*, and *R*- factors based on ALL data will be even larger.
Geometry. All e.s.d.'s (except the e.s.d. in the dihedral angle between two l.s. planes) are estimated using the full covariance matrix. The cell e.s.d.'s are taken into account individually in the estimation of e.s.d.'s in distances, angles and torsion angles; correlations between e.s.d.'s in cell parameters are only used when they are defined by crystal symmetry. An approximate (isotropic) treatment of cell e.s.d.'s is used for estimating e.s.d.'s involving l.s. planes.
Refinement. Refinement of *F*^2^ against ALL reflections. The weighted *R*-factor *wR* and goodness of fit *S* are based on *F*^2^, conventional *R*-factors *R* are based on *F*, with *F* set to zero for negative *F*^2^. The threshold expression of *F*^2^ > σ(*F*^2^) is used only for calculating *R*-factors(gt) *etc*. and is not relevant to the choice of reflections for refinement. *R*-factors based on *F*^2^ are statistically about twice as large as those based on *F*, and *R*- factors based on ALL data will be even larger.

### Fractional atomic coordinates and isotropic or equivalent isotropic displacement parameters (Å^2^)

**Table:**

	*x*	*y*	*z*	*U*_iso_*/*U*_eq_	
O	1.1101 (2)	0.21659 (16)	0.30550 (13)	0.0497 (3)	
N1	0.7461 (2)	0.13586 (16)	0.41052 (13)	0.0369 (3)	
H1A	0.6695	0.0611	0.3982	0.044*	
N2	0.4263 (2)	0.20173 (15)	0.63054 (13)	0.0353 (3)	
C1	0.6435 (2)	0.23152 (17)	0.54787 (14)	0.0316 (3)	
C2	0.7512 (3)	0.35023 (19)	0.59089 (16)	0.0393 (3)	
H2B	0.9066	0.3632	0.5336	0.047*	
C3	0.6200 (3)	0.4479 (2)	0.72110 (18)	0.0434 (4)	
H3A	0.6853	0.5296	0.7523	0.052*	
C4	0.3907 (3)	0.42384 (19)	0.80528 (17)	0.0422 (3)	
H4A	0.2982	0.4900	0.8924	0.051*	
C5	0.3035 (3)	0.29915 (19)	0.75631 (16)	0.0381 (3)	
H5A	0.1507	0.2816	0.8138	0.046*	
C6	0.9524 (2)	0.14708 (17)	0.29436 (15)	0.0322 (3)	
C7	0.9716 (2)	0.06764 (16)	0.14401 (14)	0.0290 (3)	
C8	1.2030 (2)	−0.03051 (17)	0.06322 (14)	0.0314 (3)	
H8A	1.3391	−0.0509	0.1057	0.038*	
C9	1.2324 (2)	−0.09833 (17)	−0.08032 (15)	0.0321 (3)	
H9A	1.3876	−0.1641	−0.1340	0.039*	

### Atomic displacement parameters (Å^2^)

**Table:**

	*U*^11^	*U*^22^	*U*^33^	*U*^12^	*U*^13^	*U*^23^
O	0.0418 (6)	0.0697 (7)	0.0464 (6)	−0.0318 (5)	−0.0005 (5)	−0.0194 (5)
N1	0.0417 (6)	0.0460 (6)	0.0291 (5)	−0.0261 (5)	−0.0008 (5)	−0.0092 (5)
N2	0.0366 (6)	0.0411 (6)	0.0302 (5)	−0.0166 (5)	−0.0041 (4)	−0.0045 (4)
C1	0.0368 (6)	0.0353 (6)	0.0256 (6)	−0.0148 (5)	−0.0065 (5)	−0.0023 (5)
C2	0.0426 (7)	0.0445 (8)	0.0364 (7)	−0.0224 (6)	−0.0055 (6)	−0.0067 (6)
C3	0.0532 (9)	0.0383 (7)	0.0443 (8)	−0.0182 (6)	−0.0115 (7)	−0.0100 (6)
C4	0.0491 (8)	0.0360 (7)	0.0382 (7)	−0.0074 (6)	−0.0055 (6)	−0.0110 (5)
C5	0.0360 (7)	0.0401 (7)	0.0353 (7)	−0.0101 (6)	−0.0029 (5)	−0.0041 (5)
C6	0.0332 (6)	0.0359 (6)	0.0298 (6)	−0.0144 (5)	−0.0052 (5)	−0.0039 (5)
C7	0.0300 (6)	0.0323 (6)	0.0259 (6)	−0.0139 (5)	−0.0030 (4)	−0.0015 (4)
C8	0.0267 (6)	0.0384 (7)	0.0308 (6)	−0.0119 (5)	−0.0068 (5)	−0.0011 (5)
C9	0.0263 (6)	0.0359 (6)	0.0320 (6)	−0.0084 (5)	−0.0020 (5)	−0.0057 (5)

### Geometric parameters (Å, °)

**Table:**

O—C6	1.2169 (16)	C4—C5	1.377 (2)
N1—C6	1.3614 (16)	C4—H4A	0.9300
N1—C1	1.4034 (16)	C5—H5A	0.9300
N1—H1A	0.8600	C6—C7	1.5009 (17)
N2—C1	1.3375 (17)	C7—C8	1.3888 (17)
N2—C5	1.3403 (17)	C7—C9^i^	1.3936 (17)
C1—C2	1.3932 (18)	C8—C9	1.3860 (17)
C2—C3	1.378 (2)	C8—H8A	0.9300
C2—H2B	0.9300	C9—C7^i^	1.3936 (17)
C3—C4	1.384 (2)	C9—H9A	0.9300
C3—H3A	0.9300		
			
C6—N1—C1	127.85 (11)	N2—C5—C4	123.77 (13)
C6—N1—H1A	116.1	N2—C5—H5A	118.1
C1—N1—H1A	116.1	C4—C5—H5A	118.1
C1—N2—C5	117.11 (11)	O—C6—N1	124.94 (12)
N2—C1—C2	123.38 (12)	O—C6—C7	120.89 (11)
N2—C1—N1	112.88 (11)	N1—C6—C7	114.16 (11)
C2—C1—N1	123.70 (12)	C8—C7—C9^i^	119.81 (11)
C3—C2—C1	117.84 (13)	C8—C7—C6	118.37 (11)
C3—C2—H2B	121.1	C9^i^—C7—C6	121.72 (11)
C1—C2—H2B	121.1	C9—C8—C7	120.42 (12)
C2—C3—C4	119.75 (13)	C9—C8—H8A	119.8
C2—C3—H3A	120.1	C7—C8—H8A	119.8
C4—C3—H3A	120.1	C8—C9—C7^i^	119.77 (11)
C5—C4—C3	118.06 (13)	C8—C9—H9A	120.1
C5—C4—H4A	121.0	C7^i^—C9—H9A	120.1
C3—C4—H4A	121.0		

Symmetry codes: (i) −*x*+2, −*y*, −*z*.

### Hydrogen-bond geometry (Å, °)

**Table:**

*D*—H···*A*	*D*—H	H···*A*	*D*···*A*	*D*—H···*A*
N1—H1A···N2^ii^	0.86	2.34	3.1679 (15)	163

Symmetry codes: (ii) −*x*+1, −*y*, −*z*+1.
